# The Evolution of In Vitro Toxicity Assessment Methods for Oral Cavity Tissues—From 2D Cell Cultures to Organ-on-a-Chip

**DOI:** 10.3390/toxics13030195

**Published:** 2025-03-08

**Authors:** Alexandra Jităreanu, Luminița Agoroaei, Ioana-Cezara Caba, Florina-Daniela Cojocaru, Liliana Vereștiuc, Mădălina Vieriu, Ioana Mârțu

**Affiliations:** 1 Department of Toxicology, Faculty of Pharmacy, “Grigore T. Popa” University of Medicine and Pharmacy Iasi, 700115 Iasi, Romania; ioana-cezara.caba@umfiasi.ro; 2 Department of Biomedical Sciences, Faculty of Medical Bioengineering, “Grigore T. Popa” University of Medicine and Pharmacy Iasi, 700115 Iasi, Romania; florina.cojocaru@umfiasi.ro (F.-D.C.); liliana.verestiuc@bioinginerie.ro (L.V.); 3 Department of Analytical Chemistry, Faculty of Pharmacy, “Grigore T. Popa” University of Medicine and Pharmacy Iasi, 700115 Iasi, Romania; madalina.vieriu@umfiasi.ro; 4 Department of Dental Technology, Faculty of Dental Medicine, “Grigore T. Popa” University of Medicine and Pharmacy Iasi, 700115 Iasi, Romania; ioana.martu@umfiasi.ro

**Keywords:** cytotoxicity, genotoxicity, oxidative stress, 3D oral tissue models, tooth-on-a-chip

## Abstract

Since the oral cavity comes into contact with several xenobiotics (dental materials, oral hygiene formulations, drugs, or tobacco products), it is one major site for toxicity manifestation. Multiple parameters are assessed during toxicity testing (cell viability and proliferation, apoptosis, morphological changes, genotoxicity, oxidative stress, and inflammatory response). Due to the complexity of the oral cavity environment, researchers have made great efforts to design better in vitro models that mimic natural human anatomic and functional features. The present review describes the in vitro methods currently used to investigate the toxic potential of various agents on oral cavity tissues and their evolution from simple 2D cell culture systems to complex organ-a-chip designs.

## 1. Introduction

Toxicity evaluation is an essential research area, especially in the medical and pharmaceutical world. The last decades have seen a change in the approach. Ethical and economic factors caused an inclination towards developing new and accurate alternative methods (in vitro, in silico) that are in accordance with the 3R principle (reduce, refine, and replace animal use). The non-animal methods have become increasingly sophisticated over the last years, stimulated especially by the progress in the tissue engineering field [1].

Oral cavity tissues are frequently exposed directly to various xenobiotics (dental materials, oral healthcare products, drugs, tobacco products, or other agents). Therefore, the development of relevant and predictive toxicity assessment methods is essential. The purpose of this literature review is to make an extensive presentation on the main in vitro techniques currently used for oral tissue toxicity evaluation.

## 2. Materials and Methods

The literature search was conducted on different electronic databases (PubMed, Web of Science, Scopus, Google Scholar) using keywords like “cytotoxicity”, “cell viability assays”, “apoptosis”, “genotoxicity”, “oxidative stress”, “inflammatory markers”, “2D cell cultures”, “3D cell cultures”, and “organ-on-a-chip”. Only studies that used normal human oral cell cultures, 3D, or organ-on-a-chip models reflecting oral cavity structures were considered. Original articles, reviews, and book chapters published between 1990 and now were included.

## 3. Two-Dimensional Cell Culture Testing

Cell culture testing still remains one of the main techniques that can be used in toxicity assays to screen the cellular response to chemical agents. When it comes to the cytotoxicity of different xenobiotics on oral cavity tissues, several types of cells can be used, for example, human oral keratinocytes and human oral fibroblasts. Cellular toxicity testing can be performed in various ways, and the design of a study is adapted depending on the characteristics of the tested materials, the type of products, or the clinical use scenario. According to the ISO 10993-5 guideline [2], there are three types of cytotoxicity test methods: the elution test (extract dilution), the direct contact test, and the indirect contact test. The direct contact test is a more sensitive method and can detect even a weak cytotoxic potential [3]. Compared with the indirect contact method, where the tested material is placed on a cell monolayer protected by an agar coat, in the case of direct contact, the cells are more vulnerable to mechanical impairment [4]. During the elution tests, extracts of the test article are used to assess the biocompatibility and cytotoxicity of compounds leached from the tested material. Preincubated cell layers are exposed to the obtained extracts, or freshly suspended cells are seeded directly in the resulting solution. When the laboratory routine is established, there are many parameters that can influence extraction tests, and researchers must evaluate them, in order to design the best experiment for cytotoxicity evaluation (e.g., the extraction medium, the extraction ratio, the extraction temperature or pH, the extraction time, and if the extraction is performed in a static or dynamic mode) [5].

As Srivastava et al. pointed out, the type of cytotoxicity method used can greatly influence the final results. They demonstrated that the extract dilution method or the indirect contact method was not appropriate to accurately assess the safety of perfluoro-octane, a derivative used intraoperatively in vitreoretinal surgery, where it comes in direct contact with the neuroretina. The direct contact method was better for providing meaningful data regarding cytotoxicity [3]. When it comes to oral cavity tissues, the concern for selecting the best cytotoxicity method is applicable especially to dental materials that come into contact with oral structures, like root canal sealers [6,7,8].

Various biological aspects can be targeted by in vitro toxicity assays, like cell viability and proliferation, morphological changes, genotoxicity, markers of oxidative stress, or inflammatory response.

### 3.1. Cell Viability and Cell Proliferation Assays

The most investigated aspect that reflects cytotoxicity is assessing the influence of the tested agents on cell viability. These tests have many advantages. They are low-cost, allow the assessment of a large number of samples, and give reliable and reproducible results. However, in order to obtain a clear picture of the phenomenon, a researcher must use different categories of assay. A decrease in cell viability can be the result of a cytostatic effect (the inhibition of cell metabolism or proliferation) or a cytotoxic effect of a xenobiotic (actual cell death). Colorimetric assays alone, used to evaluate cell viability via metabolic activity, are not enough to support statements about cell death induction, and additional experimental approaches must be used to detect dying cells. Furthermore, another aspect that needs investigation is the elucidation of the molecular mechanism involved in cell death (apoptosis, necrosis, or autophagy) [9].

**Figure 1:**
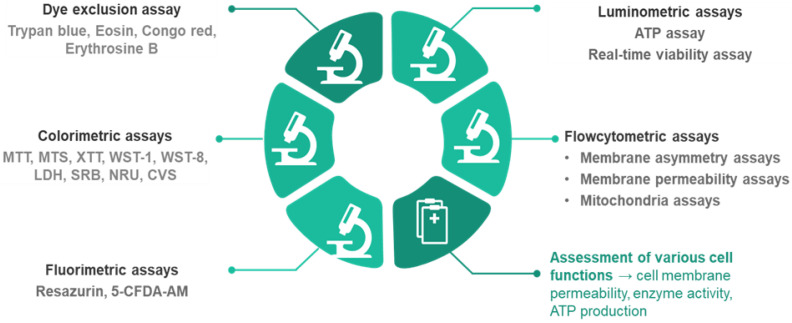
Cell viability assays.

Several methods can be used to determine the number of viable cells (Figure 1). Cell viability assays evaluate different cell functions (the permeability of cell membranes, cellular enzyme activity, ATP (adenosine triphosphate) production, and cell adherence) [10,11].

Dye exclusion assays using trypan blue, Congo red, or erythrosine B determine the integrity of the cell membranes, as only the viable cells can exclude dyes. Although these tests are fast and convenient, there are some factors that may alter the results. For example, the integrity of the membranes may not be immediately affected in lethally damaged cells. Because intracellular damage mechanisms occur before the lysis of cellular membranes, other assays can be more sensitive and accurate. Also, in some cases, the continuous proliferation of the surviving cells may underestimate cell death and cytotoxicity. Furthermore, these methods do not allow the distinction between normal, healthy cells and cells that have lost cell functions and are lethally damaged, but are still alive. Using these assays, apoptosis and necrosis processes cannot be distinguished either [12,13].

**Figure 2:**
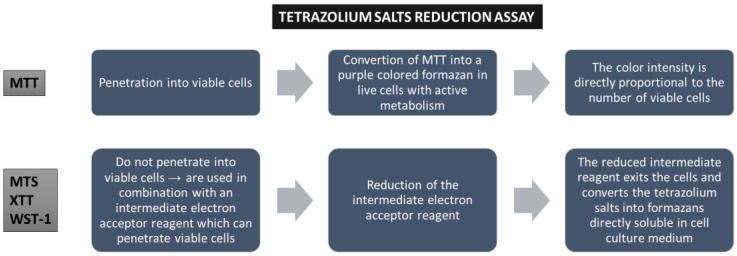
Cell viability assays based on tetrazolium salt reduction.

Colorimetric assays (MTT, MTS, XTT, and WST) evaluate cell viability via metabolic activity assessment. These assays are based on the conversion of a reagent (a specific dye) to a colored product in metabolically active cell populations, via mitochondrial enzymes. The tetrazolium salts are converted into colored formazan crystals, and the spectrophotometric signal becomes an indicator for cellular metabolic activity, which is related to the number of viable cells (Figure 2). WST (water-soluble tetrazolium) salts (WST-1 and WST-8) represent a new generation of reagents that are more stable and give a more efficient spectrophotometric signal. These methods are widely used, mainly because they are inexpensive, but their sensitivity is limited, the reagents are toxic to cells, and they are unsuitable for long-term studies. Furthermore, the distinction between cytotoxic and cytostatic effects is not possible [13,14].

The neutral red assay is based on the capacity of viable cells to incorporate the dye (neutral red) in lysosomes, and the reduction in uptake is associated with a lower cellular viability and cytotoxicity [15].

Sulforhodamine B assay is used to determine cell density, based on the measurement of cellular protein content. The cells are fixed with trichloroacetic acid and the sulforhodamine B dye binds to the basic amino acid residues of cellular proteins. The amount of protein-bound dye, determined spectrophotometrically, is directly proportional to the number of living cells [13,16].

Fluorometric methods (e.g., alamarBlue) were developed as an alternative to colorimetric methods, and have the advantages of a higher sensitivity. The alamarBlue assay is suitable for long-term experiments, due to the lower degree of toxicity. These assays are based on the conversion of nonfluorescent agents into fluorescent compounds by cellular enzymes (e.g., viable cells reduce resazurin to resorufin, a highly fluorescent compound). The fluorescent signal is proportional to the percentage of viable cells with an active cellular metabolism [11]. 5-CFDA-AM (5-carboxyfluorescein diacetate acetoxymethyl ester) is a cell-permeant esterase substrate that can be used for the same purpose, as it evaluates both enzymatic activity and cell-membrane integrity. Intracellular esterases activate fluorescence, while the integrity of cellular membranes is a condition for intracellular retention of the fluorescent product.

Another colorimetric assay is LDH. The method assesses cellular membrane integrity by measuring the level of lactate dehydrogenase released from the cytoplasm of affected cells into the cell culture medium after membrane disruption in necrotic or apoptotic cells. The quantitative determination of the enzyme is achieved via the conversion of tetrazolium salt into red formazan. The major limitation of this method is that it requires low-serum or serum-free conditions [12,13].

The glucose-6-phosphate dehydrogenase (G6PD) release assay is also a method based on the quantification of an enzyme released from dead cells via damaged membranes into the culture medium, but it is more sensitive than the LDH assay. It is a fluorescence-based assay, involving the reduction of resazurin to resorufin, in the presence of NADPH, generated by G6PD [13].

One of the fastest and most sensitive methods to evaluate cell viability is the ATP assay, a luminometric assay. This method is based on the transformation of luciferin to oxyluciferin, a reaction catalyzed by luciferase in the presence of Mg ions and ATP. The intensity of the luminescent signal depends on the levels of ATP synthesized by viable cells [10,12]. The major drawback of this method is the impossibility of distinguishing between cytostatic and cytotoxic effects because several factors can influence the ATP level (cell proliferation, cell death, or metabolic activities) [13]. One luminometric assay allows monitoring of cellular viability in real time (the real-time viability assay). It uses an enzyme (luciferase) and a prosubstrate. Live cells internalize the prosubstrate and transform it into an active substrate that interacts with luciferase and generates a bright signal. The main advantage is the ability to observe both dose- and time-dependent toxicity on the same set of samples, as continuous readings can be performed over extended periods [17].

Flow cytometry has become a versatile tool for qualitative and quantitative cell analysis. Flow cytometry has wide applications, including toxicity studies, because it is a rapid and reliable method to distinguish between viable and dead cell populations. This method reveals information about cells’ size and granularity, but it also allows fluorescent staining targeting various cellular parameters. One of these parameters is the composition of the outer surface of the cell membrane. In the early stages of apoptosis, this composition is altered, even while cells preserve their membrane integrity. Consecutively, phosphatidylserine can be detected at the outer surface of cells by fluorescent labeling with Annexin V. Another way to assess membrane asymmetry is using F2N12S, a molecule with dual-color fluorescence emission that is incorporated into the phospholipids of cell membranes. Apoptosis causes alterations of the cellular membrane charges, and the color of the emission is influenced by these changes, allowing the determination of the viability percentage [11,18]. Other flow cytometric assays use dyes to assess membrane integrity and permeability (inclusion dyes, exclusion dyes, and monomeric cyanine nucleic acid stains) or they determine various mitochondria parameters (mitochondria membrane potential, mitochondria mass, and mitochondria membrane permeability) [11].

The flow cytometry-based Annexin V assay is capable of distinguishing between different types of cell death (apoptosis vs. necrosis) in a single population of cells. In necrotic cells, the membrane becomes permeable to propidium iodide, a DNA-binding fluorescent dye, while apoptosis is detected using Annexin V, which binds to the phosphatidylserine [19]. For precise discernment between apoptosis and necrosis, the levels of specific biomarkers and regulators can be assessed using Western blotting [e.g., the activation of caspase-3 and PARP1 (poly (ADP-ribose) polymerase-1) for apoptosis; RIPK1 (receptor-interacting serine/threonine kinase 1) phosphorylation for necrosis]. Imaging flow cytometry can also be used to analyze the morphological differences between apoptotic and necrotic cells [9].

Cell proliferation is another parameter that characterizes cells’ health and the dynamic of the cell population. Cellular division plays a significant role in physiological and pathological processes and is usually investigated not only in studies concerning malignancies and anticancer therapy, but also in toxicity assays [13].

The clonogenic cell survival assay investigates the capacity of cells to retain their ability to proliferate and form a colony (or a clone). This method has the advantage of considering reversible damage induced by exposure to xenobiotics, and the regrowing phenomenon of resistant cells. However, the clonogenic (colony formation) assay is not suitable for agents that do not affect DNA synthesis; it is applicable only to adherent cells, it is time-consuming, and in the case of manually counting the colonies, errors may appear [13,20].

Other common assays include the detection of mitotically active cells and direct measurement of DNA synthesis via the incorporation of thymidine analogs—BrdU (5-bromo-2′-deoxyuridine) or EdU (5-ethynyl-2-deoxyuridine)—into nascent DNA during S-phase. The newly synthesized BrdU-incorporating DNA can be detected immunologically with a specific antibody against BrdU [13,21,22]. Another approach to labeling nascent DNA is using the incorporation and detection of tritium-labeled (H3) thymidine, but due to the difficulties in handling radiolabeled substances, the BrdU/EdU methods are preferred [13]. Cell proliferation can also be analyzed using the detection of proliferation markers (e.g., proliferating cell nuclear antigen (PCNA), Ki67 antigen, and phosphohistone H3 (PHH3)). PCNA confers high processivity to DNA polymerase delta and has a major role in DNA replication, but it is also involved in many other important cellular processes (cell cycle control, DNA repair, epigenetic inheritance) [23]. Ki67 is a nuclear antigen expressed in all proliferating cells (both normal and cancerous), in all phases of the interphase (G1, S, and G2) and mitosis. The Ki67 index (percentage of Ki67-positive cells) becomes an excellent marker for cell proliferation because the antigen is not only expressed in cells during the resting state (G0 phase). The quantification protocols imply the use of specific Ki67 antibodies, and the detection methods can be flow-cytometry or a colorimetric approach [13].

Cytotoxicity studies can also be performed with real-time cell analyzers that allow dynamic live-cell monitoring and electronic detection of biological processes. This approach offers continuous information about cell adhesion, growth and proliferation, morphological changes, and cell death [24,25].

Although these tests are widely used, there are several factors that can induce variability in response and affect reproducibility. These factors are biological (e.g., cells and medium characteristics) or technical (e.g., counting errors, the edge effect, treatment time, or cross-reactivity with other compounds). A suboptimal experimental design leads to significant errors, and it is very important to optimize the experimental parameters [26].

Table 1 presents a selection of studies investigating the cytotoxic potential of different materials and chemical agents on various types of normal oral cavity cell cultures using cell viability and proliferation assays.

Apoptosis assays

**Figure 3:**
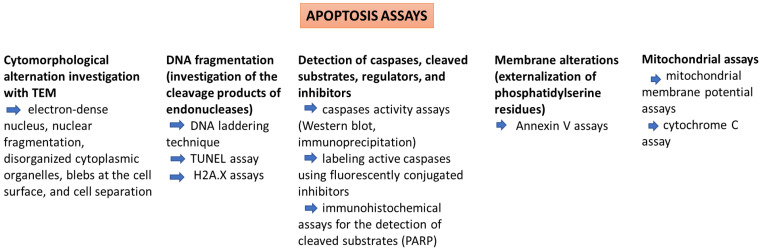
Apoptosis assays.

Traditionally, cell death induced by cytotoxic agents is associated with necrosis, but xenobiotics can promote toxicity by triggering apoptosis. Apoptosis appears as a defense mechanism in cells damaged by noxious agents [72]. Several methods can be used for identifying and quantifying apoptosis (Table 2). Morphological assessment is one of them, because there are typical alterations that accompany apoptosis (e.g., cell shrinkage, denser cytoplasm, tightly packed organelles, membrane blebbing, and the formation of apoptotic bodies). Apoptosis assays also evaluate other key cellular events (Figure 3) that occur during programmed cell death, such as DNA fragmentation (TUNEL assay), activation of the caspase-dependent proteolytic cascade (caspase activity assay), and the assessment of the expression levels of apoptotic proteins (Western blot analysis) [73].

During apoptosis, the endonucleases cause the fragmentation of nuclear DNA and nuclear destruction. The TUNEL (Terminal deoxynucleotidyl transferase-mediated dUTP Nick End Labeling) assay allows the in situ identification of DNA strand breaks in apoptotic nuclei by fluorochrome-labeling of free 3′-hydroxyl termini of DNA strand breaks. However, other deoxynucleotides (instead of dUTP) can also be used in different variants of this assay. The cells can be analyzed by flow cytometry or laser scan cytometry (for cells attached to microscope slides). False TUNEL positivity can be determined by high DNA repair rates in proliferating or necrotic cells [74].

Caspases (cysteine-aspartic proteases) are endopeptidases that are synthesized in an inactive form and are later activated via proteolytic cleavage. These enzymes are activated as a response to cytotoxic agents, which eventually leads to the disruption of cellular processes and cell death. Caspases can be considered potential markers of apoptosis caused by cell exposure to toxins [75]. Caspases can be assessed using different methods. Colorimetric assays are based on the spectrophotometric detection of chromophores (e.g., p-nitroanilide) cleaved by the enzyme from the labeled substrates. On the other hand, fluorometric assays use fluorochrome substrates (e.g., 7-amino-4-trifluoromethyl coumarin) and the fluorescent signal is quantified [76]. Other techniques used to detect the activation of caspases are the Western blot assay which uses an antibody specific to active caspase, immunocytochemical localization of an activated caspase with specific antibodies, and the FLICA assay (using Fluorochrome-Labeled Inhibitors of Caspases) [77].

**Table 2:** Selection of studies assessing apoptosis of human oral cells during cytotoxicity testing.

Method	Type of Cells	Tested Agent	Results	Reference
TUNEL assay	Human gingival fibroblasts	Hydrogen peroxide	Hydrogen peroxide promoted apoptosis in human gingival fibroblasts.	[78]
	Oral mucosa epithelial cells	Mobile phone use (radiofrequency radiation)	Apoptosis levels were not significantly influenced.	[79]
	Human gingival fibroblasts	Nicotine	Nicotine increased the number of TUNEL-positive cells.	[80]
	Human gingival epithelium keratinocytes	Traditional cigarette smoke and electronic cigarette aerosols	Exposure to traditional cigarette smoke and electronic cigarette aerosols increased the number of TUNEL-positive cells.	[39]
	Human oral fibroblasts	Cigarette smoke and e-vapor condensates	The DNA fragmentation assay indicated an increased number of TUNEL-positive apoptotic fibroblasts with broken nuclei.	[40]
	Human gingival epithelial cells	E-cigarette vapor	E-cigarette vapor exposure increased the proportion of TUNEL-positive cells.	[58]
Caspase activity assay	Human gingival fibroblasts	Nicotine	Nicotine induced apoptosis via the caspase-3 pathway.	[80]
	Human gingival epithelial cells	Cigarette smoke total particulate matter (TPM), smokeless tobacco extracted with complete artificial saliva (ST/CAS), and whole-smoke conditioned media (WS-CM).	TPM and WS-CM (combusted tobacco) significantly increased caspase-3 activity, while ST/CAS (non-combusted) showed no or only minimal activation.	[55]
	Human gingival epithelial cells	E-cigarette vapor	A significant induction of caspase-3 proteins was observed in e-cigarette vapor-exposed cells.	[58]
	Human gingival fibroblasts	E-cigarette vapors and cigarette smoke	No significant differences were recorded for the caspase 3/7 activity in the tested groups.	[81]
	Odontoblast-like MDPC-23 cells	NaF	NaF exposure induced the activation of caspase-3.	[82]
	Cementoblasts	Zinc-oxide eugenol-based root-canal sealer	The root-canal sealer stimulated caspase-3, -8, and -9 activity in a dose-dependent manner.	[76]
	Human oral fibroblasts	Antioxidant mixtures of resveratrol (R), ferulic acid (F), phloretin (P), and tetrahydrocurcuminoids (T)—RFT, PFR, and PFT	The tested mixtures did not increase apoptosis.	[43]
	Human gingival fibroblasts	Epicatechin gallate	Epicatechin gallate did not induce caspase-3 activity.	[52]
Image-iTTM LIVE green caspase detection	Human oral fibroblasts	Antioxidant mixtures of resveratrol (R), ferulic acid (F), phloretin (P), and tetrahydrocurcuminoids (T)—RFT, PFR, and PFT	The tested mixtures did not increase apoptosis.	[43]
DAPI staining and microscopy analysis	Human gingival fibroblasts	Nicotine	Apoptotic cellular bodies, nuclear condensation, and DNA fragmentation were present.	[80]
	Human gingival epithelium keratinocytes	Traditional cigarette smoke and electronic cigarette aerosols	Apoptotic morphology was detected; there were no significant differences in cell morphology between t-cigs and e-cigs.	[39]
Annexin- V-FITC	Human gingival fibroblasts	Commercial topical fluoride varnishes	Some of the tested samples exhibited high cytotoxicity, associated with certain components present in the fluoride varnishes (cetylpyridinium chloride and ethyl acetate).	[83]
	Human normal oral epithelial cells	Cisplatin and thymoquinone (alone and in combination)	Thymoquinone (in combination with cisplatin) decreased cisplatin-induced apoptosis and necrosis in normal cells.	[49]
	Human gingival fibroblasts	Vitamin C	Vitamin C reduced apoptosis rates in human gingival fibroblasts exposed to *Porphyromonas gingivalis*.	[84]
	Human gingival fibroblasts	Curcumin	The proportion of apoptotic cells increased with increasing concentrations of curcumin.	[85]
	Human oral fibroblast	Menthol and eucalyptol	The results indicated a concentration-dependent and exposure-time-dependent cytotoxicity for eucalyptol and menthol.	[86]
	Human gingival fibroblasts	Methacrylate-based resins	Methacrylate-based resins produced cell death via necrosis or apoptosis, depending on the sample.	[87]
	Human gingival fibroblasts	N-acetylcysteine incorporated in methacrylate-based resin cement	N-acetylcysteine diminished cytotoxicity, cell apoptosis, and necrosis in cells.	[88]
	Human gingival fibroblast	Dental resin polymerization initiators	Dental resin polymerization initiators induced cell death via necrosis.	[89]
Western blot analysis of apoptotic proteins	Human gingival fibroblasts	Nicotine	Nicotine increased BAX expression (pro-apoptotic protein) and decreased BCL-2 expression (anti-apoptotic protein).	[80]
	Human normal oral epithelial cells	Cisplatin and thymoquinone (alone and in combination)	Cisplatin and thymoquinone (alone and in combination) showed a significant upregulation of p53 and caspase-9 (pro-apoptotic), and a decrease in the anti-apoptotic Bcl-2 protein expression.	[49]
	Human gingival fibroblasts	Hydrogen peroxide	H_2_O_2_ increased the BAX expression and decreased the Bcl-2 expression.	[78]
Mitochondrial membrane potential assay	Human gingival fibroblasts	Curcumin	Curcumin induced early apoptotic events characterized by the loss of the mitochondrial membrane potential.	[85]
	Human gingival fibroblasts	TEGDMA released from resin composite	TEGDMA induced mitochondrial damage and mitochondrial membrane potential collapse.	[90]

Abbreviations: BAX (Bcl-2-associated X protein); F (ferulic acid); P (phloretin); R (resveratrol); ST/CAS (smokeless tobacco extracted with complete artificial saliva); T (tetrahydrocurcuminoids); TEGDMA (triethyleneglycol dimethacrylate); TPM (cigarette smoke total particulate matter); and WS-CM (whole-smoke conditioned media).

### 3.2. Morphological Changes Assessment

Cell morphology describes the shape, size, and internal structure of cells and is considered a visual indicator of the cellular homeostasis and physiological state of cells. There is substantial evidence that cellular compartments change in response to external factors, and these modifications are accompanied by alterations in cellular functions. The interactions between the extracellular environment and the cells influence highly regulated biological processes and affect the cell shape, size, and texture of cellular compartments. Therefore, cell morphological profiling has become a valuable tool for toxicity assays, offering important clues about the intracellular targets and the toxicity mechanism [91,92,93]. Furthermore, morphological identification of cell death is more accurate than other methods and can be considered the golden standard. Microscopy analysis (e.g., SEM) completes and supports the results of cell viability assays and biochemical analysis [94]. In the -omics era, a new term has emerged. Morphomics is used to emphasize the importance of cell morphology. The morphome (the totality of the cells’ morphology parameters) is determined by molecular dynamics (genome, transcriptome, proteome, and metabolome) [95].

The main limitation of cell morphological profiling consists of the difficulty of scaling up, because, conventionally, the examination of every image was performed manually. However, the development of high-content screening systems may represent an opportunity for the emergence of assays based on cell morphological profiling, suitable for cytotoxic screening. Computational tools are able to analyze vast quantities of data and project a quantitative and statistically valid relationship between cell shape and experimental conditions [93]. The current trend is to use artificial intelligence approaches to analyze cellular images [95]. The REM-I platform is a new technology that uses artificial intelligence to analyze and extract phenotypic features of cells and sort them according to their morphology from high-resolution images [96,97].

The current advances in the field of microscopy and computer image analysis have brought this research area to an impressive level. For example, the “Cell Painting” assay represents an image-based cell profiling technique based on the fluorescent labeling of major cellular organelles that allows the detection of cellular morphological features in response to different treatments. Usually, six fluorescent stains are used, over five channels [actin + Golgi + plasma membrane (AGP), nucleus (DNA), endoplasmic reticulum (ER), nucleoli + cytoplasmic RNA (RNA), mitochondria (Mito)], imaging eight internal cellular structures and organelles [98].

Over 1500 morphological features can be acquired at the single-cell level using this assay, giving information about cellular functions alteration. This phenotypic profiling allows the investigation of the biological impact of molecular or genetic perturbations in a relatively unbiased manner, unlimited by preconceived notions or existing knowledge [99,100]. This high-throughput phenotypic profiling assay is approved for bioactivity screening and chemical risk assessment by the Center for Computational Toxicology and Exposure and the United States Environmental Protection Agency, due to its broad applicability [101,102].

Cell painting allows researchers to study cell viability, cell proliferation, DNA damage, cell cycle stage, and the dynamic organization of proteins [103]. Some modified Cell painting experiments allow not only the detection of some morphological features, but also the quantitative assessment of phenomena like apoptosis, oxidative stress, or autophagy, using fixable functional markers. Small-molecule sensor dyes were developed to detect caspase activation in cell cultures or mitochondrial superoxide generation associated with cell stress using fluorescence microscopy [104].

Fluorescence imaging techniques have been used in studies involving human oral cells [105,106,107], but, to our knowledge, no research papers using the Cell Painting assay on these types of cells have been published so far.

Several features are analyzed in morphological profiling (differences in size, shape, compactness, organelle symmetry, staining intensities, textural patterns, and spatial organization of cells. The most common morphological changes are usually cell detachment and shape alteration, due to cytoplasmatic shrinkage [108].

The morphological changes that appear in the internal cellular structure can be seen at different levels (cytoplasm, cytoskeleton, nucleus, Golgi apparatus, and endoplasmic reticulum). The alterations that affect the cell cytoplasm are among the most obvious, and Lai et al. developed a cytotoxicity assay using cytoplasm-localized fluorescent probes (CLFPs). The authors use fluorescent staining to differentiate between living and dead cells. Due to the differences in the permeability of the plasma/nuclear membrane, the live cells appear with a bright cytoplasm and a dark nucleus, while the dead cells are fully fluorescent. Dead cells without intact membranes will not be visible by fluorescence microscopy. Some companies have developed assays designed to assess changes in the morphological profile of cells by evaluating the nuclear area with a high content screening (HCS) system [94].

When it comes to assessing the cytotoxicity of materials and chemical compounds on human oral cavity cells, the most frequently tested agents can be divided into three main categories—dental materials, drugs used locally or for the treatment of different systemic diseases, and other agents/xenobiotics (Table 3).

### 3.3. Genotoxicity Assessment

**Figure 4:**
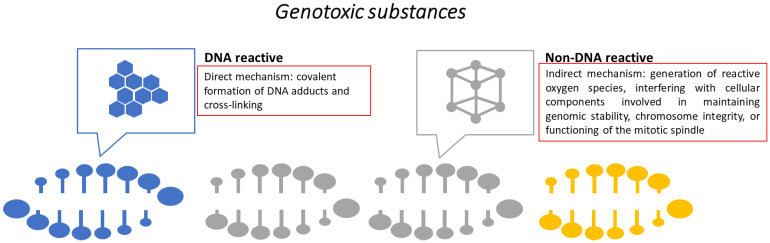
Genotoxicity mechanisms.

Another important aspect investigated is the genotoxic potential. Genotoxic substances alter the structure, information content, and segregation of DNA. Mutagenic agents permanently modify the genetic material in the cell. There are several mechanisms involved in genotoxicity (Figure 4) and batteries of broad in vitro tests (e.g., bacterial reverse mutation assay, in vitro chromosomal aberration test, micronucleus test, comet assay, and DNA damage and repair) [119,120]. In vitro genotoxicity assays are robust and simple tools, both time- and cost-effective, with a low rate of false negative results, due to their high sensitivity. One downside of these assays is their relatively low specificity, which is associated with a high rate of false positive results. Therefore, the challenge is to develop assays with adequate sensitivity and improved specificity [120].

For in vitro genotoxicity testing on human oral cells, the assays most commonly used are the micronucleus test (an assay for chromosomal abnormalities), the comet assay (a test for primary DNA damage), and the H2AX assay (Table 4).

Due to its convenience and reliability, the micronucleus assay has become a popular tool to assess genotoxicity. Micronuclei represent round-shaped extranuclear bodies, chromatin-containing, that are visible in the cytoplasm, with different origins (acentric chromosome fragments, malsegregation of chromosomes, dicentric chromosome breakage, and chromosome instability). They are considered a marker for DNA damage and genomic instability. A version of this assay is the cytokinesis-block micronucleus assay, which uses cytochalasin to stop cytokinesis (but not karyokinesis), and the micronuclei are scored only in binucleated cells. This version has the advantage of fewer false negative results [119,121].

The comet assay evaluates single or double DNA breaks, cross-linking, damage to DNA bases, and apoptotic fragments in isolated cells using gel electrophoresis. The DNA damage and relaxation of the supercoiled DNA will cause the differential migration of damaged and undamaged DNA during electrophoresis, stretching toward the anode, and forming a comet-like structure [122].

The H2AX assay reflects double-strand breaks. DNA damage and the formation of double-strand breaks (DSBs) are followed by the phosphorylation of H2AX, a component of the histone octamer in nucleosomes. A newly phosphorylated protein, γ-H2AX, is formed, which can be considered a biomarker for DSBs. γ-H2AX can be detected using specific monoclonal or polyclonal antibodies. The overall γ-H2AX levels in cell lysates can be determined by the ELISA (enzyme-linked immunosorbent assay), while the measurement of γ-H2AX levels directly in cell nuclei can be achieved by microscopy or fluorescence-activated cell sorting (FACS). The counting of γ-H2AX foci is a very sensitive marker of DNA damage, and to increase efficiency, high throughput foci counting systems were developed [119,123,124,125].

**Table 4:** Selection of studies assessing genotoxicity on human oral cells.

Method	Type of Cells	Tested Agent	Results	Reference
Micronucleus assay	Oral epithelial cells	Liquids for electronic cigarettes	Positivity for micronuclei was recorded for flavored and unflavored e-liquids.	[54]
	Human oral keratinocytes	Dental photoinitiators (BAPO and TPO)	No genotoxic effect was observed.	[30]
	Human gingival fibroblasts	Single-wall carbon nanotubes	The frequency of micronuclei was higher after exposure to lower doses (50–100 μg/mL), and lower for high-dose treatments (125–150 μg/mL).	[41]
	Human pulp fibroblasts	*Aloe vera* associated with endodontic medication (calcium hydroxide) and laser photo-biomodulation	The highest micronucleus rate was obtained for the association *Aloe vera* + laser therapy.	[126]
	Human gingival fibroblasts	Toothpastes	The tested toothpastes did not exhibit genotoxic effects.	[127]
	Human gingival fibroblasts	Static magnetic field produced by dental magnetic attachments	The micronuclei rates increased in the exposed groups, but no mitotic changes appeared.	[128]
	Human gingival fibroblasts	*Achyrocline satureioides* extract	The micronucleus count increased in a concentration-dependent manner; no genotoxic effects were recorded for concentrations up to 6.25 mg/mL.	[129]
	Human gingival fibroblasts	Methacrylic monomer BAPP	A dose-related increase in the number of micronuclei was recorded.	[130]
Comet assay	Human gingival fibroblasts	Single-wall carbon nanotubes	The nanotubes induced a significant increase in tail moments.	[41]
	Human gingival fibroblasts	Bracket alloys	All samples exhibited genotoxicity, but the Ni-Ti combination showed the highest DNA damage.	[131]
	Human gingival fibroblasts and buccal epithelial cells	Corrosion eluates from stainless-steel brackets, nanoparticle-coated brackets, and polymeric-coated brackets	The stainless-steel bracket eluates caused increased nuclear damage in cells.	[132]
	Human gingival fibroblasts	Metals released from three orthodontic alloys (stainless-steel, nickel-free, and titanium)	The greatest DNA damage was produced by the stainless-steel alloy.	[133]
	Human gingival keratinocytes	Corrosion eluates produced from orthodontic materials	No apparent DNA damage was recorded.	[134]
	Human oral keratinocytes	TEGDMA	The increase in tail moments after TEGDMA exposure was concentration-dependent.	[135]
	Human gingival fibroblasts	BAPP	The incidence of DNA strand breaks increased in a concentration-dependent manner.	[130]
	Human gingival fibroblasts	HEMA	HEMA induced significant fragmentation of the DNA.	[136]
	Human gingival fibroblasts	Non-irradiated campherquinone	The exposure to campherquinone caused significant DNA damage.	[137]
	Oral epithelial cells	Liquids for electronic cigarettes	No significant increase in DNA damage assessed by the comet assay was recorded.	[54]
	Human periodontal ligament fibroblasts	E-cigarette vapors with flavoring	An increase in fluorescent tail length was observed, indicating DNA damage.	[138]
γH2AX assay	Human gingival fibroblasts	HEMA	A significant extent of DNA double-strand breaks was observed.	[136]
	Human gingival fibroblasts	Methacrylate-based adhesives	The highest level of DNA damage induction was exhibited by the adhesive with diphenyliodonium hexafluorophosphate as a co-initiator.	[46]
	Human gingival fibroblasts	N-acetylcysteine incorporated in methacrylate-based resin cement	Incorporating N-acetylcysteine resulted in an anticytotoxic effect.	[88]
	Human gingival fibroblasts	Methacrylate-based monomers in association with antioxidants (N-acetylcysteine and ascorbic acid)	The addition of antioxidants reduced the number of DNA double-strand breaks after exposure to methacrylate-based monomers.	[125]
	Human gingival fibroblasts	Titanium dioxide-modified glass ionomer cements	No genotoxic effects were observed for the titanium dioxide-modified glass ionomer cements.	[139]
	Human gingival fibroblasts	Dental resin restoration components (TEEGDMA, Neopen, DPIC, TPSB, TPP)	The induction of DNA double-strand breaks in exposed cells was observed, with the highest incidence for diphenyliodoniumchloride and neopentylglycol dimethacrylate.	[140]
DNA precipitation assay	Buccal fibroblasts	Arecoline	No DNA single-strand breaks appeared after exposure to arecoline.	[141]
Integrity of genomic DNA (electrophoresis)	Human gingival fibroblasts and buccal epithelial cells	Stainless-steel brackets, nanoparticle-coated brackets, and polymeric-coated brackets exposed to corrosion eluates	The number of cellular alterations was insignificant.	[132]
	Human gingival fibroblasts	Silver tungstate microcrystals	DNA integrity was not affected.	[62]

Abbreviations: BAPO (phenyl-bis(acyl) phosphine oxide); BAPP (2,2-bis[4-(acryloxypropoxy) phenyl] propane); DPIC (diphenyliodonium chloride); HEMA (2-hydroxylmethyl methacrylate); Neopen (neopentylglycol dimethacrylate); TEEGDMA (tetraethyleneglycol dimethacrylate); TEGDMA (triethyleneglycol dimethacrylate); TPO (diphenyl(acyl) phosphine oxide); TPP (triphenylphosphane); and TPSB (triphenyl-stibane).

### 3.4. Oxidative Stress

Another mechanism involved in oral tissue damage is oxidative stress. The oral cavity is considered a part of the human body that is especially susceptible to oxidative damage, as there are many sources of free radicals in the oral environment (food, xenobiotics, microorganisms, dental materials and treatments, and local inflammatory processes). The saliva contains both enzymatic and nonenzymatic antioxidants (e.g., superoxide dismutase, catalase, glutathione peroxidase, salivary peroxidase, transferrin, and lactoferrin) [142]. Oxidative stress is defined as the disruption of the balance between the free radicals produced (reactive oxygen and nitrogen species) and the capability of antioxidant systems to counteract them. Oxidative damage affects all cellular components (lipids, proteins, and nucleic acids), and can lead to oral and systemic diseases [143]. Oxidative stress is a key element involved in the progression of periodontal diseases and chronic inflammation. Free radicals’ overproduction is associated not only with the loss of periodontal tissue integrity, but also with morphological alterations in the salivary glands, or with the appearance of precancerous lesions or oral cancers [142].

Animal and cell cultures have been used to study oxidative stress effects and antioxidant mechanisms. Still, experiments conducted on live animals are more difficult to interpret due to the complexity of tissues and organs’ organization and functioning. Therefore, cell culture models are widely used as models of oxidative stress [144]. One method to evaluate oxidative stress is to determine the intracellular levels of free radicals by evaluating the intracellular oxidation of 2′,7′-dichlorodihydrofluorescein diacetate (H2DCF) to 2′,7′-dichlorofluorescein (DCF) and measuring fluorescence by flow cytometry [145]. Other assays focus on assessing the antioxidant defense system [the concentration of reduced glutathione, the activity, and the expression of antioxidant enzymes (e.g., glutathione peroxidase, catalase, and heme oxygenase)]. The Western blot assay, a method based on polyacrylamide gel electrophoresis, is frequently used to assess antioxidant enzymes [144]. In order to maintain a stable intracellular redox state, the expression of these enzymes is modified as an adaptive cell response in an attempt to maintain the homeostatic concentrations of reactive oxygen species (ROS). The expression of several antioxidant enzymes becomes a reflection of the pro-oxidant–antioxidant balance and oxidative stress and can be measured using qRT-PCR (quantitative real-time PCR) [145].

**Figure 5:**
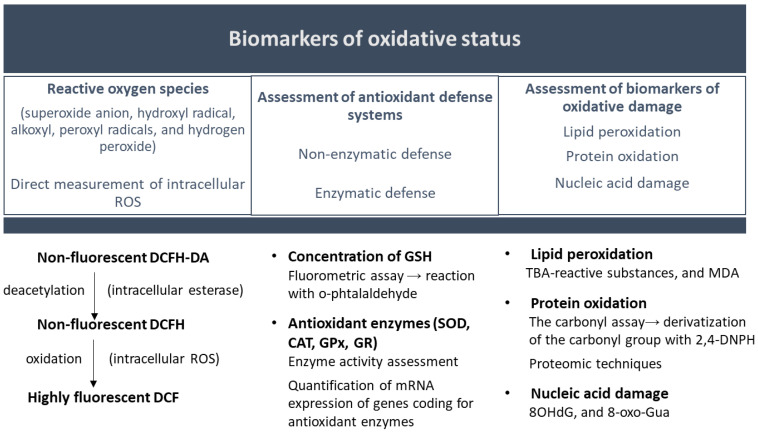
Oxidative status assessment. Abbreviations: CAT (catalase); DCF (2′,7′-dichlorofluorescein); DCFH-DA (dichloro-dihydro-fluorescein diacetate); DNPH (2,4-dinitrophenylhydrazine); GPx (glutathione peroxidase family); GR (glutathione reductase); GSH (glutathione); MDA (malondialdehyde); SOD (superoxide dismutase); TBA (thiobarbituric acid); 8OHdG (8-hydroxy-2′-deoxyguanosine); and 8-oxo-Gua (8-oxoguanine).

The high reactivity and the short half-life of reactive oxygen species make their quantification difficult. Under these circumstances, the evaluation is based on the quantification of biomarkers of oxidative damage to molecules (e.g., malondialdehyde for lipid peroxidation, 8-hydroxy-2′-deoxyguanosine for DNA oxidative damage, or protein carbonylation) (Figure 5) [144,146,147].

Several techniques can be used to determine protein carbonylation. The simplest and most accessible method is the Levine spectrophotometric assay, based on the chromogenic reaction between carbonyl groups and 2,4-dinitrophenylhydrazine. 2,4-dinitrophenylhydrazine derivatization of protein carbonyls is also used in other techniques, like High Performance Liquid Chromatography (HPLC), immunoblotting (using anti-DNP antibodies), or ELISA. Although it does not give quantitative data, the immunoblot detection of carbonyl groups (Oxyblot) is used especially in cell culture studies due to its sensitivity and specificity. However, if a more detailed insight into the mechanism of protein oxidative damage is required, proteomic techniques are necessary [147,148].

Dental adhesives are involved in the induction of oxidative stress. Several research teams studied the effects on cellular redox homeostasis of photoinitiators for photo-polymerizable dental resinous materials. Conventionally, camphorquinone (CQ) is used, but there are also alternatives (e.g., phenyl-bis(acyl) phosphine oxide (BAPO) and diphenyl(acyl) phosphine oxide (TPO)). The researchers assessed the oxidative stress in oral cells by the quantification of the intracellular formation of reactive oxygen/nitrogen species (ROS/RNS) or by determining the mRNA expression levels of oxidative stress genes and discovered that the intracellular free-radical-generating activity of BAPO and TPO was significantly lower compared to CQ [30,145].

Biocompatibility and toxicity associated with dental alloys used in restorative dentistry have represented popular research topics. Several corrosion products can be released in the oral cavity, and this phenomenon can affect the health of the human oral tissues. Metal ions are frequently produced via corrosion, and one of the cellular effects is the increased formation of ROS and oxidative stress. Lee et al. studied the effects of indium ions on oral keratinocytes by detecting the intracellular ROS formation. Intracellular production of reactive oxygen species is a complex process, with several pathways being involved (the mitochondrial respiratory cycle, the NADPH oxidase, and the xanthine-oxidase pathways). Furthermore, increased production of ROS can influence the terminal differentiation of oral keratinocytes, a vital process in preserving the barrier of oral mucosa [47].

Other examples of studies investigating cellular redox homeostasis in oral cells are presented in Table 5.

### 3.5. Inflammatory Response

Inflammation is a natural phenomenon that can be induced by various factors (infectious, physical, chemical, and autoimmune), but when it is extended or prolonged, can lead to cellular damage. Inflammatory response assessment in host cells is a key element for biocompatibility and toxicity evaluation because it can represent the starting point for toxic effects and cell damage [153].

Cytokines are soluble factors, proteins with a low molecular weight, involved in orchestrating the inflammatory response, and their quantification represents an indicator of the cellular inflammatory status. There are several categories of cytokines: tumor necrosis factors (TNFs), interleukins (ILs), lymphokines, monokines, interferons (IFNs), colony-stimulating factors (CSFs), and transforming growth factors (TGFs). Some cytokines are pro-inflammatory, while others are anti-inflammatory [154]. IL-6 is a pro-inflammatory cytokine produced by various oral cell types during the early stages of inflammation, but its dysregulated synthesis can initiate a chronic inflammatory status. Prostaglandins (e.g., PGE2) are synthesized by the cyclooxygenases COX-1 and COX-2 from arachidonic acid. COX-2 overexpression causes the cyclooxygenase pathways of inflammation [155,156]. Furthermore, activated oral cells can also secret matrix metalloproteinases (MMPs), proteolytic enzymes whose overexpression and uncontrolled release and activation can lead to inflammation and tissue destruction [157].

To assess inflammatory responses, several markers (e.g., pro-inflammatory cytokines interleukins, TNF-α, and IFN-γ) can be quantified using ELISA, but transcriptional analysis can also evaluate the expression levels of key genes associated with cellular inflammation (Table 6) [33].

## 4. Three-Dimensional Cell Culture Models

Although 2D cell culture models are still the most commonly used tools for in vitro studies, they exhibit several limitations. As 2D cell culture systems partially fail to replicate the architecture and physiology of living tissues, 3D models were designed to simulate better in vivo conditions (cell differentiation, gene and protein expression, and cell-to-cell communication). In monolayered cultures, the tested compounds must be dissolved in the aqueous culture medium since the cell cultures are submerged. This inconvenience disappears for 3D models cultured at the air–liquid interface; with cells placed into cell culture inserts, tissue feeding is achieved via a basal microporous membrane, and the apical tissue surface is not submerged in culture medium, which allows the direct application of xenobiotics [167,168]. Another disadvantage that appears when monolayer cell cultures are used in toxicity and exposure risk assessment studies is the direct access of xenobiotics to the proliferating cells, in contrast with in vivo systems that present an epithelial barrier [168].

Different three-dimensional oral models, histologically and biologically similar to human oral tissues, have been developed since 1997, and have been used to determine the oral mucosal irritation and cytotoxicity of oral care products and dental materials, but also to study drug delivery via the oral mucosa, oral pathology, and response to tobacco products or UV radiation (Table 7) [168].

Two types of 3D models can be developed—full- and split-thickness models. The split-thickness models comprise only the epithelium barrier (keratinocytes placed directly on permeable membranes at the liquid–air interface) and are usually used for permeation studies. The full-thickness models include a connective tissue layer beside the epithelial layer, and, usually, the connective layer represents a 3D collagen scaffold, infiltrated with fibroblast. These full-thickness models more effectively recapture the complex structural and functional characteristics of the native tissue [169].

AlFatlawi et al. published a comprehensive systematic review regarding the use of 3D gingival and mucosal models in periodontal research. They concluded that there is a lack of standardization regarding the fabrication and characterization of 3D systems and a high degree of heterogeneity among models. According to their study, most researchers used rat tail collagen as substrates for the construction of the models, but bovine collagen, acellular cadaveric dermis, and porcine collagen were also utilized. Primary human gingival keratinocytes and fibroblasts, or immortalized cell lines, were used as the cellular source, and the number of epithelial cell layers varied between 4 and 16. Most models were constructed under static cell culture conditions. The authors emphasized the importance of developing a standardized characterization protocol to confirm the validity of 3D oral models, one that includes histological confirmation and evidence of the differentiation of tissue layers using specific markers [167].

Some models are currently commercially available: the human oral mucosal epithelium models [EpiOralTM (MatTeK Corporation, Ashland, MA, USA) and SkinEthic Human Oral Epithelium (Episkin, Lyon, France)] and the human oral gingival epithelium models [EpiGingivalTM (MatTeK Corporation, Ashland, MA, USA) and SkinEthic Human Gingival Epithelium (Episkin, Lyon, France)] [170,171]. DentCytoTool is a 3D dentin/pulp tissue analog used for the cytotoxicity and biocompatibility assessment of dental restorative materials. It contains two compartments: the dentin/odontoblastic layer (the upper compartment) and the lower compartment representing a pulp analog [172].

**Table 7:** Selection of studies using 3D models for toxicity assessment.

Type of 3D Model	Investigated Parameter	Tested Agent	Results	Reference
Dental materials and oral healthcare formulations				
Human gingival epithelial culture model EpiGingival™	Cell viability and retention of o-cymen-5-ol and zinc	Toothpaste with o-cymen-5-ol and zinc	No cytotoxic effect appeared and the delivery of o-cymen-5-ol and zinc was successful.	[173]
Human oral epithelial culture model EpiOral™	Tissue viability and cytokine release	Prototype oral care formulations	The models were useful to screen the irritation effect of oral products.	[174]
Three-dimensional human oral mucosal model	Histology, tissue viability, and IL-1β release	Dental composite resins	TEGDMA-based composite resin increased IL-1β release and caused mucotoxicity.	[175]
Human oral epithelial culture model EpiOral™	Tissue viability, histology, and caffeine permeability	Caffeine, ethanol, and mouthrinses	Ethanol-containing mouthwashes did not exhibit a cytotoxic effect on the mucosal tissue; the permeability of caffeine was not affected.	[176]
Human oral epithelial culture model EpiOral™	Viability, cytokine release, levels of protein markers for apoptosis, DNA damage, cell proliferation, cell–cell adhesion	Mucoadhesive polymer blend (pullulan, tamarindus indica polysaccharide, and sodium hyaluronate)	No cytotoxic effect was observed and the cytokine levels were not modified; there was no indication of DNA damage.	[177]
Reconstituted human oral epithelial tissue models (3D) (SkinEthic Laboratories)	Cell viability and morphology	Point-welded, laser-welded, and silver-soldered orthodontic wires	Viability and histological evaluation did not reveal signs of severe toxicity.	[178]
Oral mucosa tissue model (3D)	Transepithelial electrical resistance, cell viability, and cytokine secretion	Mouth rinsing solutions	Cell viability was not affected, but only one solution had no impact on TEER. One product increased IL 8 secretion.	[179]
DentCytoTool	Viability, cytotoxicity, and odontogenesis- and angiogenesis-related markers	HEMA; TEGDMA	The cytotoxic potential increased in a time- and concentration-dependent manner, and the expression of angiogenesis-related markers was reduced; the LPS/TEGDMA co-treatment aggravated the cytotoxic effects.	[172]
Pulp analogue (3D)	Cell viability/proliferation, morphology, and expression of angiogenic and odontogenic markers	Calcium-silicate-based cements	The materials were biocompatible, with positive angiogenic and odontogenic effects.	[180]
Oral mucosal models	Cytotoxicity, histological, and permeation analyses	Mucoadhesive patches with clobetasol-17-propionate	No tissue damage was recorded.	[181]
Nicotine, ENDS, and tobacco products				
Human oral epithelial culture model EpiOral™	Cytotoxicity, histological analysis, cytochrome P450 activity, production of pro-inflammatory mediators, and transcriptomics	Cigarette smoke and aerosols obtained from a modified-risk tobacco product	Cytotoxicity, morphological alterations, production of inflammatory mediators, and transcriptomic changes were significantly more pronounced for cigarette smoke.	[182]
Human gingival epithelial culture model EpiGingival™	Cytotoxicity, histological analysis, cytochrome P450 activity, production of pro-inflammatory mediators, transcriptomics, and metabolomics	Cigarette smoke and aerosols obtained from a modified-risk tobacco product	The aerosol from the modified-risk tobacco product caused minimal cytotoxicity and minor histopathological alterations, and had a low impact on transcriptomic and metabolomic data.	[183]
Human gingival epithelial culture model EpiGingival™	Pro-inflammatory cytokine levels, inflammation and DNA damage markers (RAGE, COX-2, and γH2A.X)	E-cigarette aerosols	E-cigarette exposure augmented inflammation and DNA damage markers.	[138]
Human oral epithelial culture model EpiOral™	Viability, morphology, and tissue absorption	E-cigarette aerosol	No decrease in viability and no change in morphology and tissue structure were observed.	[184]
Human oral epithelial culture model EpiOral™ Human gingival epithelial culture model EpiGingival™	Cytotoxicity (adenylate kinase assay), histology, inflammatory mediator secretion, transcriptomics, and targeted proteomics	Cigarette smoke and a carbon-heated tobacco-product aerosol	The carbon-heated tobacco-product aerosol exhibited a significantly lower biological impact in comparison to cigarette smoke.	[185]
Human gingival epithelial culture model EpiGingival™	Irritant potential	Extracts from nicotine pouch products	The tested products were not irritants.	[186]
Oral mucosa tissue model (3D) with normal fibroblasts and cancerous TR146 keratinocytes	Histological examination and viability	E-cigarette liquids	Prolonged exposure and high concentrations of e-liquids caused cytotoxic effects for normal cells but stimulated the growth of cancerous cells.	[187]
Other agents				
Gingival culture system (3D)	DNA analysis	Sodium butyrate	Sodium butyrate induced DNA release in a time- and dose-dependent manner, and stimulated the release of SAP130.	[188]
Human oral epithelial culture model EpiOral™	Cell viability and histological analysis	Sodium lauryl sulfate	EpiOral is a suitable system for oral mucosal irritation test.	[170]
Human gingival epithelial culture model EpiGingival™ Human oral epithelial culture model EpiOral™	DNA damage, CPD repair rate, and apoptotic cell numbers	UV radiation	UVB radiation presented a higher carcinogenic risk for oral tissues.	[189]
Human gingival epithelial culture model EpiGingival™ Human oral epithelial culture model EpiOral™	Cytokine release, DNA damage, CPD repair rate, and apoptotic cell numbers	UVB radiation	Increased interleukin-8 (IL-8) release, low rates of CPD repair, and decreased apoptotic cell numbers.	[190]
Cell culture model (3D) (gingival fibroblasts in collagen matrix)	Cell viability and morphology, and gene expression of growth factors	Low-level laser therapy	Low-level laser therapy increased viability and promoted biostimulation of gingival fibroblasts.	[191]
Reconstituted human oral epithelial tissue models (3D) (EpiSkin Laboratories)	Cell viability and morphology	Carrageenan	No loss of viability was recorded for the carrageenan-treated sample.	[31]
Human gingival epithelial culture model EpiGingival™	Cytotoxicity, viability, histology, immunohistochemistry (Ki67, VEGF-A), and TUNEL assay	Low-temperature plasma	Low-temperature plasma had low cytotoxicity and high cellular viability.	[192]
Human gingival epithelial culture model EpiGingival™	Viability	Lipophilic vehicles	No signs of cytotoxicity were recorded.	[193]

Abbreviations: COX-2 (cyclooxygenase-2); CPD (cyclobutane pyrimidine dimer); γH2A.X (phosphorylated form of the histone variant H2AX); LPS (lipopolysaccharides); RAGE (receptor for advanced glycation end); SAP130 (Sin3A-associated protein 130 kDa); TEGDMA (triethyleneglycol dimethacrylate); TEER (transepithelial electrical resistance); and VEGF-A (vascular endothelial growth factor-A).

As presented in Table 7, the assessment of toxicity and biocompatibility using 3D oral models can be performed using the same parameters as in 2D cell culture studies (cell viability and proliferation, morphological changes, genotoxicity assessment, oxidative stress, and inflammatory response).

### 4.1. Cell Viability

Evaluating cell viability and proliferation remains a key aspect of cytotoxicity assessment for 3D cellular systems.

Most studies performed on 3D oral models have used the MTT assay to assess the metabolic activity of the cellular population and cytotoxicity [173,176,178,179,180,181,184,186,193]. The MTT assay also allows indirect measurement of the xenobiotics’ effect on the barrier function of the tissue [174]. Other researchers have used resazurin-based assays to evaluate cell viability (the alamarBlue assay [175] and PrestoBlue assay [187]), the advantages being the lack of toxicity of the dye (allowing continuous monitoring) and the greater sensitivity of detection. The quantification of lactate dehydrogenase (LDH assay) [193] and the adenylate kinase test (which detects ATP generated from ADP and the final product is determined colorimetrically or fluorometrically) [182,183,185] were also used for the evaluation of cell viability. To evaluate the safety of a mucoadhesive polymer blend, cell proliferation markers PCNA, Cyclin A, and Cyclin D1 were determined in EpiOral tissue models [177].

However, the transition of the standardized viability assays from 2D to 3D systems can be challenging due to the structural differences and the more sophisticated architecture of the 3D models, leading to inaccurate results and misleading conclusions [194].

Dominijanni et al. investigated the accuracy of cell viability assays in 3D hydrogel systems. They pointed out that indirect viability assays (the MTS assay, PrestoBlue fluorometric assay, CellTiter-Glo luminescent assay, and measuring ATP) developed for 2D monolayer cultures presented significant differences when used in 3D hydrogels. Several factors can influence performance, like the construct size, the hydrogels’ formulation, porosity, and the diffusion process, causing some reagents or metabolites to become trapped in the 3D construct, affecting the final results. The direct viability methods (BrU incorporation and FACS—fluorescence-activated cell sorting) give more reliable results. Direct evaluation via histological techniques (live/dead assay by fluorescence microscopy) offers a good alternative, the stained histological sections indicating healthy and apoptotic cells and their spatial distribution in the 3D model, but it is time-consuming. To obtain the best results, indirect viability assays can be validated via direct microscope techniques [195].

### 4.2. Morphological Changes

Exposure to xenobiotics can alter the tissue morphology, and for 3D models, this aspect can be analyzed using a histological examination with light microscopy after performing hematoxylin and eosin staining. Tissues (epithelia) react to harmful and irritating agents through morphological adaptations, while highly toxic compounds cause tissue damage. Various parameters can be investigated: cell morphology, the continuity and thickness of the epithelium, keratinization, the aspect of the connective tissue layer, and the presence of pyknotic nuclei [187].

Signs of morphological alteration of the human oral and gingival epithelial culture model (hyperkeratinization, keratohyalin granules, and the accumulation of nuclei in the stratum corneum) were visible after exposure to cigarette smoke [182,183,185].

### 4.3. Genotoxicity Assessment

The International Workshop on Genotoxicity Testing Working Group (IWGT WG), one of the leading groups in regulatory genotoxicology, has analyzed the topic of genotoxicity testing using 3D models and agreed that 3D systems are more relevant than 2D assays to evaluate human genotoxicity because the 3D models are based on human cells. Three-dimensional assays are more difficult to perform, more expensive, and time-consuming, but they also address all the endpoints of genotoxicity mentioned for 2D cell culture systems (mutagenicity, clastogenicity, and aneugenicity). So far, 3D-skin comet and micronucleus assays have gained sufficient validation to support the development of individual OECD Test Guidelines [196].

Three-dimensional oral and gingival models also proved useful for genotoxicity assessment. Sundar et al. evaluated the DNA-damaging effect of e-cigarette vapors using the comet assay and γ-H2AX levels [138]. Furthermore, to assess the genotoxicity of UV radiation for oral tissues, Agrawal et al. and Breger et al. determined DNA damage (cyclobutane pyrimidine dimers), the repair rate of cyclobutane pyrimidine dimers, and the number of apoptotic cells [189,190].

### 4.4. Oxidative Stress and Inflammatory Response

In vitro studies of toxicological processes using 3D oral models can also investigate the induced oxidative stress and inflammatory response.

Transcriptomics and metabolomics can reveal the changes induced by oxidative stress. The adaptative response is reflected in the alteration of the expression of genes involved in the reactive oxygen species pathway, but also in the perturbation of the metabolite profile, in an attempt to counteract the oxidative challenge. For example, cigarette smoke influenced the perturbation of metabolites in the glutathione pathway [182,183].

An increase in cytokines, chemokines, and growth factors released from oral epithelial models can be a response to exposure to dental materials, oral hygiene formulations, or other xenobiotics. To quantify the secreted inflammatory mediators [tumor necrosis factor α (TNFα), colony-stimulating factor (CSF2, CSF3), interleukin (IL-6, IL-8, IL-1A, IL-1B), chemokine (CXCL8, CXCL10), matrix metalloproteinase (MMP-1, MMP-9), and vascular endothelial growth factor alpha (VEGFA)], the ELISA technique [175,179] or Luminex xMAP technology can be used, which allows simultaneous identification and quantitation via a flow-based methodology that uses a dual-laser system and color-coded beads with unique spectral codes. The inflammatory response is also visible in the changes that appear in the expression of inflammation-related genes [182,183].

To sum up, 3D oral tissue models are valid and accurate tools for in vitro studies and present many advantages when compared to 2D monolayer cultures, and also to animal models. The structures and functions of oral human native tissues are closely reproduced, and some issues associated with animal testing (e.g., species extrapolation and ethical problems) are avoided when using tissue models.

## 5. Organ-on-a-Chip Systems

The continuous search for more human-relevant in vitro models is directed towards the development of models that not only can capture and recreate organ structures and tissue–tissue interfaces using multiple cell layers, but also mimic the functionality of an organ. Organ-on-a-chip devices are in vitro micro-physiological systems, and, so far, represent the best in vitro tools for toxicity prediction [197,198,199,200].

These devices use microfluidic technology to allow human cells and tissues to be cultured under dynamic fluid flow. They recreate the architecture of an organ but also mimic vascular perfusion, interstitial flow, and the complex interactions that occur in live tissues. Furthermore, these systems can replicate the immune response by circulating immune cells through the vascular endothelium-lined channel, and the complex living microbiome can be co-cultured with human cells. This is a big step forward since there is substantial scientific evidence that supports the important role that the microbiome has in both human physiological and pathological processes [197,199].

Since the beginning of the 21st century, when microfluidic technology was adapted for the development of in vitro organ models, multiple organ-on-a-chip systems were developed, covering almost all the organs in the human body (lung, liver, kidney, gut, skin, brain/blood–brain barrier, bone/bone marrow, fat, muscle, and retina) [200].

This technology was also used in dental research, although the organ-on-a-chip field is underdeveloped in this research domain compared to other areas. However, different types of microphysiological systems have been designed and used so far in dental and oral research. One-chamber design chips, with a unique culture chamber connected to channels for the transport of fluids, are usually used to simulate the impact of the oral environment (e.g., salivary flow) on biofilm formation. Parallel-chamber chips, with two or more parallel chambers interconnected, are the most used models to simulate the architecture of natural tissues and are usually used in cytotoxicity testing [201,202].

Research conducted to evaluate the oral toxicity of different agents has pointed out that in some monolayer cell culture experiments, the cytotoxic effect was overestimated due to the direct contact between the tested compounds and oral cells. The oral cavity environment is complex, influenced by processes like salivary flow and microbial colonization, with the gingival epithelium and dentin acting as barriers against chemicals and toxins. Furthermore, dental tissues have a unique characteristic, combining soft (dental pulp) and mineralized tissues (dentin, enamel), which poses extra challenges for accurate in vitro testing. Organ-on-a-chip models offer an alternative closer to in vivo conditions and actual clinical practice [203,204,205].

The first attempts to recreate the natural oral environment date back to the 1980s, when barrier models for dental pulp studies were designed. Today, the dentin barrier cytotoxicity test (ISO 7405 [206]) is used to assess the biocompatibility of dental materials. At the beginning of the 21st century, a new ex vivo model was developed, an entire human tooth culture, but the static conditions (pH and CO_2_/O_2_ exchange) were its main limitations [205].

Regarding toxicity testing on oral cavity cells, the biomaterials used for dental treatment occupy first place in biocompatibility and toxicity assessment. França et al. were the first to develop a “tooth-on-a-chip”. They designed a microfluidic chip of the pulp–dentin–biomaterials interface to test the cytotoxicity of dental materials on pulp cells. The chip has the great advantage of closely recapturing the physiological microenvironment at the pulp–dentin interface and enabling live-cell imaging to study the morphological profile of dental pulp cells exposed to dental materials [207].

Zhang et al. constructed a dentin-on-a-chip device using microfluidic chip technology by encapsulating stem cells from the apical papilla in gelatin methacrylate [208]. Muniraj et al. created a gingiva-on-a-chip model with a multi-chamber design and a vertically stacked configuration of polymethyl methacrylate sheets. Primary human gingival fibroblasts and human oral keratinocytes were used as cell cultures and upper and lower channels were created to deliver media, air, chemicals, or nutrients and to collect the metabolic waste [204]. Jin et al. designed a gingival epithelium–capillary interface-on-a-chip to study the inflammation of the periodontal soft tissue and the response to therapeutic agents [209].

There are several advantages of using organ-on-a-chip models as in vitro testing and monitoring tools. The main advantage include the ability to obtain cellular structures and dynamic experimental conditions similar to in vivo ones. Regarding oral and dental research, different scenarios of mechanical stress were recreated using these models (e.g., orthodontic forces, periodontal ligament–alveolar bone interface environment, or gradient flow in gingival crevices). Furthermore, these micro-sized culture chambers allow cost-effective parallelization for high-throughput experiments [201].

To assess morphological changes, cell viability, and proliferation of cell cultures on-chip, the technology currently used is light-phase and fluorescence imaging. The design of the organ-on-a-chip systems is made from optically transparent materials and is compatible with direct visualization in real time. Therefore, live/dead staining can be used to detect cellular viability. H–E (hematoxylin and eosin)-stained images give information regarding the integrity of various structures (e.g., the gingival epithelial layer), some of the histological parameters investigated being desquamation, cellular vacuolization, or loss of intercellular cohesion [203,207,209]. Furthermore, fluorescent dyes or antibodies can be used to evaluate the expression of certain biomarkers (e.g., immunostaining was used to observe the expression of the proteins F-actin and VE-cadherin) [209]. Makkar et al. also evaluated the tissue viability depending on the lactate dehydrogenase (LDH) release for his “gingival crevice-on-a-chip” microfluidic platform [210].

The organ-on-a-chip models allow the collection of media and the quantification of different biomarkers, evaluating the inflammatory response of the host tissue. Makkar et al. observed that microfluidic devices better mimic in vivo inflammatory events because, under traditional static experimental conditions, an over-estimation of the innate immune response can occur [210].

Examples of studies using organ-on-a-chip systems for toxicity and biocompatibility are presented in Table 8.

## 6. In Vitro Models for Toxicity Assessment: Advantages, Challenges, and Future Perspectives

Studying cell populations is an essential aspect of research in many domains, from drug discovery, toxicity assessment, cancer research, or other areas of research trying to offer some insight into other pathological mechanisms. Two-dimensional cell cultures have been used for decades and are valuable, simple, and inexpensive screening tools with multiple applications and highly replicable results, suitable for large-scale studies. However, as a monolayer of cells grown on a plate, the natural environment is not recreated; cell proliferation is unnaturally fast, and differentiation is poor. Furthermore, testing drug response or toxicity can be inaccurate due to higher substance-induced apoptosis rates [214].

With 3D cell culture systems, the environment better mimics the in vivo conditions in terms of cell shape, differentiation and proliferation rates, as well as the expression levels of genes and proteins. Nevertheless, there are disadvantages associated with this technique. It is more expensive and time-consuming, the experiments are harder to replicate, and some assays established for 2D cultures must be validated for 3D models to ensure correct and accurate results [214].

As the techniques for tissue engineering have advanced, organ-on-a-chip systems have emerged, models that replicate tissue functionality and represent a bridge that can cover the gap between traditional in vitro studies and animal models. They represent predictable models for human physiological and pathological processes and recreate natural barriers, being more accurate tools for assessing bioactivity and toxicity. Another advantage is the possibility for continuous real-time measurements and visualization due to the various sensors that can be adapted to the model. The main challenges associated with this technology are the costs and the need for model standardization. The current designs are characterized by a high degree of heterogeneity, making it difficult to carry out comparative analyses [198,201,215,216].

Despite the limitations of these new microfluidic devices, future advancements will probably overcome them, bringing researchers closer to achieving the “human-on-a-chip” concept.

## 7. Conclusions

The oral cavity is a major site for toxicity manifestation, as it comes into contact with several xenobiotics (dental materials, oral hygiene formulations, drugs, or tobacco products). Assessing toxicity for oral cavity tissues is challenging due to the complex structure of the tissues and the particular physiology. Most studies still apply traditional 2D cell culture systems despite their limitations. However, the emergence of new technologies (3D cell cultures and organ-on-a-chip models) offers the possibility to investigate physiological and pathological processes in a dynamic and complex environment, resembling in vivo scenarios. Continued research is directed towards overcoming the difficulties associated with these innovative techniques, leading to a rapid evolution of the in vitro methods used in this research area. We expect future research to focus mainly on organ-on-a-chip systems designed to closely mimic the anatomy and physiology of oral cavity tissues.

## Figures and Tables

**Table 1:** Selection of studies assessing cell viability and proliferation of human oral cells during cytotoxicity testing.

Method	Type of Cells	Tested Agent	Results	Reference
Dye exclusion assays				
Trypan blue dye exclusion test	Human oral keratinocytes	Biopolymer-coated liposomes (alginate, gellan gum, chitosan)	The formulations presented a low cytotoxicity; cell viability was above 70% in all cases.	[27]
	Human gingival fibroblasts	Zoledronic acid	An intense reduction in the number of viable cells was observed after exposure to zoledronic acid.	[28]
	Human dental pulp fibroblasts	Dental materials (HEMA and adhesive)	The adhesive system showed significant cytotoxic effects at a concentration of 10^−2^; cytotoxic effects decreased when the material was polymerized before testing.	[29]
Colorimetric assays				
MTT	Human oral keratinocytes	BAPO and TPO—alternative photoinitiators to CQ in dental resinous materials	BAPO and TPO presented higher cytotoxicity than CQ.	[30]
	Human salivary cell gland acinar cell line	Carrageenan	Carrageenan is safe and non-toxic to the oral cavity tissues as an anti-human papillomavirus agent.	[31]
	Non-keratinized human oral mucosa cells	ZnO, TiO_2_, SiO_2_, and hydroxyapatite (bulk particle-sized materials and nanomaterials)	ZnO (70 nm NP and bulk) caused a significant reduction in cell viability; TiO_2_ (bulk and nano form) was well tolerated by the cells.	[32]
	Human gingival stroma fibroblasts	Dental adhesives	Short-term stimulation and long-term inhibition of cell viability were observed for the undiluted extracts.	[33]
	Human gingival and oral fibroblasts	Calcite (calcium carbonate) and zincite (zinc oxide) nanoparticles	Cell viability decreased in a concentration-dependent manner, but the tested materials were regarded as non-cytotoxic.	[34]
	Rat gingival fibroblasts	Calcium hydroxide and ellagic acid preparations (different ratios)	The mixture calcium hydroxide–ellagic acid was non-toxic and promoted the proliferation of gingival fibroblasts.	[35]
	Human gingival fibroblasts	Zoledronic acid	Zoledronic acid decreased cell viability by 40%.	[28]
	Human oral fibroblasts	Acid bone lysate	The viability of human gingival fibroblasts was maintained with a 5% concentration of acid bone lysate.	[36]
	Odontoblast-like cells (MDPC-23), human dental pulp cells	H_2_O_2_ bleaching gel	The cell viability reduction was proportional to the concentration and contact time.	[37]
	Human oral mucosa fibroblasts	Ultraviolet B radiation	UVB-irradiated cells exhibited significantly reduced proliferation.	[38]
	Human gingival epithelium keratinocytes	Traditional cigarette smoke and electronic cigarette aerosols	Traditional cigarette smoke and electronic cigarette aerosols decreased cell viability; the acute toxic effect of e-cig aerosols was lower.	[39]
	Human oral fibroblasts	Cigarette smoke and e-vapor condensates	Cigarette smoke and e-vapor condensates reduced cell viability.	[40]
MTS	Human gingival fibroblasts	Single-wall carbon nanotubes	Single-wall carbon nanotubes did not affect cell viability at low concentrations but showed strong cytotoxicity at high concentrations (150 μg/mL).	[41]
	Human oral epithelial cells	Commercial resin bonding agents	Most uncured materials had significant, concentration-dependent, cytotoxic effects, while most post-cured samples revealed no cytotoxicity.	[42]
	Human oral fibroblasts	Antioxidant mixtures of resveratrol (R), ferulic acid (F), phloretin (P), and tetrahydrocurcuminoids (T)—RFT, PFR, and PFT	Low concentrations of antioxidant mixtures increased viability, but high concentrations reduced cell survival.	[43]
	Human gingival fibroblasts	Chitosan particles	Chitosan increased cell viability.	[44]
XTT	Human oral fibroblasts	Denture adhesives	The tested denture adhesives exhibited no cytotoxic effect.	[45]
	Human gingival fibroblasts	Methacrylate-based adhesives	Cell viability decreased in a concentration-dependent manner.	[46]
WST	Human oral keratinocytes	Silver–palladium–gold–indium dental alloy (In^3+^ released by corrosion)	Low concentrations of In^3+^ increased cell viability, while higher concentrations (1.6 mM) significantly decreased the percentage of viable cells and might have damaged the human oral epithelium.	[47]
	Human oral epithelial cells	Commercial resin bonding agents	Significant cytotoxic effects were exhibited by most of the uncured materials.	[42]
	Human oral keratinocytes	Biopolymer-coated liposomes (alginate, gellan gum, and chitosan)	The formulations showed cell proliferation results comparable to the control.	[27]
	Human gingival fibroblasts	Er:YAG (erbium-doped yttrium–aluminum–garnet) laser irradiation	Cellular proliferation depended on the applied energy levels, with a maximum registered at 6.3 J cm^−2^.	[48]
	Human oral epithelial cells	Cisplatin and thymoquinone (alone and in combination)	Thymoquinone presented lower cytotoxicity against normal cells in comparison to cisplatin, but the interaction between cisplatin and thymoquinone was synergistic, increasing the killing effect against normal cells.	[49]
NRU	Human gingival epithelial cell	Dental restorative materials	Transwell inserts and the NRU assay proved a reliable method to assess cytotoxicity of dental materials.	[50]
	Human gingival fibroblasts	Mouthwashes	The products presented a dose-dependent cytotoxicity but can be considered safe in diluted solutions.	[51]
	Human gingival fibroblasts	Tea polyphenols (ECG, EGCG, EC, EGC, CG, and C)	ECG and CG exhibited high toxicity, EGCG was moderately toxic, and EGC, C, and EC presented low toxicity (after a 3-day exposure).	[52]
	Human gingival fibroblasts	Theaflavin mixture purified from black tea	The theaflavin mixture exhibited cytotoxic effects only at high concentrations; normal cells were less susceptible to cytotoxicity than cancerous cells.	[53]
	Human oral epithelial cells	Liquids for electronic cigarettes	There was heterogeneity across e-liquids in terms of cytotoxicity; the presence of certain flavoring agents (ethyl maltol, vanillin, maltol, and ethyl vanillin) increased the risks.	[54]
SRB	Human gingival epithelial cell	Cigarette smoke total particulate matter, smokeless tobacco extracted with complete artificial saliva, and whole-smoke conditioned media.	Combusted products exhibited higher cytotoxicity; nicotine alone did not show significant signs of cytotoxicity in the applied range.	[55]
LDH	Human gingival fibroblasts	Novobiocin	The percentage of LDH-releasing cells increased in a concentration-dependent manner.	[56]
	Non-keratinized human oral mucosa cells	ZnO, TiO_2_, SiO_2_, and hydroxyapatite (bulk particle-sized materials and nanomaterials)	ZnO (bulk and 70 nm NP) caused a significant increase in LDH release.	[32]
	Human gingival fibroblasts	ZnO nanoparticles	MTT assay proved to be a more sensitive marker for cytotoxicity.	[57]
	Human gingival fibroblasts	Chitosan particles	ZnO NP increased cytoplasmic LDH release only at high concentrations.	[44]
	Human gingival epithelial cells	E-cigarette vapor	LDH levels were not significantly altered.	[58]
Acid phosphatase	Human oral fibroblasts	ZrO_2_ nanoparticles reinforced 3D-printed dental resins	The addition of ZrO_2_ nanoparticles did not interfere with biocompatibility; 3D-printed specimens were noncytotoxic.	[59]
Fluorimetric assays				
Resazurin-based assays (alamarBlue, PrestoBlue)	Human oral gingival epithelial keratinocytes, oral gingival fibroblasts	Long-chain bases (sphingosine, dihydrosphingosine, and phytosphingosine)	Gingival epithelial keratinocytes were more resistant to the three long-chain bases than gingival fibroblasts.	[60]
	Human gingival fibroblasts	Areca nut (*Areca catechu* L.) products	Areca nut extracts exhibited necrotizing and cytotoxic effects.	[61]
	Human gingival fibroblasts	Silver tungstate (α-Ag_2_WO_4_) microcrystals	Concentrations of 0.781 and 7.81 μg/mL of silver tungstate microcrystals were not cytotoxic.	[62]
	Human periodontal ligament fibroblasts	E-smoking liquids	The proliferation rates were reduced in a time-dependent manner; some flavors (e.g., menthol) exhibited higher cytotoxicity.	[63]
Luminometric assays				
CellTiter-Glo	Human oral epithelial cells	Commercial resin bonding agents	Most uncured materials presented cytotoxic effects; most post-cured samples revealed no cytotoxicity.	[42]
ATP assay	Human gingival fibroblasts	Novobiocin	Novobiocin decreased cell viability; ATP assay was the most sensitive assay of cell viability.	[56]
	Normal human oral keratinocytes	Electronic cigarette aerosols	The e-cig aerosols exhibited cytotoxic effects.	[64]
	Human periodontal ligament fibroblasts	E-smoking liquids	Flavors like hazelnut, lime, or menthol reduced ATP detection.	[63]
Flowcytometric assays				
Flowcytometry	Human gingival fibroblasts	Areca nut (*Areca catechu* L.) products	Areca nut products exhibited necrotizing effects.	[61]
	Human gingival epithelial cells	Cigarette smoke total particulate matter (TPM), smokeless tobacco extracted with complete artificial saliva (ST/CAS), whole-smoke conditioned media (WS-CM).	TPM caused necrosis, WS-CM did not increase the percentage of apoptotic or necrotic cells (the mechanism of cytotoxicity was different—cell cycle arrest and/or inhibition of proliferation), the low cytotoxicity of ST-CAS was confirmed.	[55]
	Human oral keratinocytes	Smokeless tobacco extract	The percentage of cells in apoptotic death increased.	[65]
	Human gingival epithelial cells	E-cigarette vapor	E-cigarette smoke promoted apoptosis and necrosis in gingival epithelial cells.	[58]
	Human gingival stroma fibroblasts	Dental adhesives	The dental adhesives inhibited fibroblasts in the G0/G1 phase and impaired their transition to G1–S; the high cell death rate was not due to apoptosis, but possibly to necrosis.	[33]
Cell proliferation assays				
Labeling with BrdU	Human oral fibroblasts	Antioxidant mixtures of resveratrol (R), ferulic acid (F), phloretin (P), and tetrahydrocurcuminoids (T)—RFT, PFR, and PFT	Antioxidant-mixture treatment induced DNA synthesis stimulation.	[43]
	Human oral fibroblasts	Cigarette smoke and electronic cigarette condensates	Fibroblast proliferation was significantly reduced after exposure to cigarette smoke and e-cig condensates.	[40]
	Human gingival fibroblasts	Chitosan particles	Chitosan promoted cell proliferation.	[44]
	Human gingival fibroblast	Cyclosporin A	Cyclosporin A significantly stimulated the proliferation of cells in a dose-dependent manner.	[66]
	Human oral keratinocytes	BAPO and TPO—alternative photoinitiators to CQ in dental resinous materials	TPO and BAPO did not exhibit toxic effects on human oral keratinocytes.	[30]
Clonogenic assay	Oral keratinocyte cell line	Radiation	Radiation impaired cellular proliferation and migration.	[67]
	Human gingival fibroblasts	Platelet-rich plasma	Platelet-rich plasma (5%) stimulated the clonogenic ability of cells.	[68]
Radiolabeled thymidine (3H-TdR)	Human oral fibroblasts	Copper chloride solution	The addition of copper chloride had no significant effect on the fibroblast proliferation rates.	[69]
	Human gingival fibroblasts	Nicotine	Nicotine significantly inhibited proliferation.	[70]
	Human oral fibroblasts	Ultraviolet B radiation	UVB-irradiated cells exhibited significantly reduced proliferation.	[38]
PCNA expression	Human gingival fibroblasts	Cyclosporin A	Cyclosporin A significantly increased PCNA levels.	[66]
	Human gingival fibroblasts	Chitosan particles	Chitosan increased PCNA protein levels.	[44]
Ki67 assay	Human gingival fibroblasts	Chitosan particles	Chitosan increased Ki67 staining.	[44]
	Human gingival fibroblasts	Er:YAG (erbium-doped yttrium–aluminum–garnet) laser irradiation	Laser irradiation increased Ki67 staining and the number of proliferating cells.	[48]
Real-time cell analyses				
	Human gingival fibroblasts	Chlorhexidine	The inhibition of human gingival fibroblast proliferation after exposure to chlorhexidine was time- and concentration-dependent.	[71]
	Human gingival fibroblasts	Monomers/comonomers in dental resin composites (BisGMA, HEMA, TEGDMA, and UDMA)	HEMA exhibited the lowest cytotoxic effect.	[25]

Abbreviations: 3H-TdR (tritiated thymidine); ATP (adenosine triphosphate); BAPO (phenylbis(acyl) phosphine oxide); BisGMA (bisphenol A-glycidylmethacrylate); BrdU (5-bromo-2′-deoxyuridine); C (catechin); CG (catechin gallate); CQ (camphorquinone); EC (epicatechin); ECG (epicatechin gallate); EGC (epigallocatechin); EGCG (epigallocatechin gallate); Er:YAG (erbium-doped yttrium–aluminum–garnet); F (ferulic acid), HEMA (2-hydroxy-ethyl-methacrylate); LDH (lactate dehydrogenase); MTS (dimethylthiazol-carboxymethoxyphenyl-sulfophenyl-tetrazolium); MTT (methylthiazolyldiphenyl-tetrazolium bromide); NRU (neutral red uptake); P (phloretin); PCNA (proliferating cell nuclear antigen); R (resveratrol); SRB (sulforhodamine B); ST/CAS (smokeless tobacco extracted with complete artificial saliva); T (tetrahydrocurcuminoids); TEGDMA (triethyleneglycol-dimethacrylate); TPM (cigarette smoke total particulate matter); TPO (diphenyl(acyl) phosphine oxide); UDMA (urethane dimethacrylate); WS-CM (whole-smoke conditioned media); WST (water-soluble tetrazolium salt); and XTT (methoxynitrosulfophenyl-tetrazolium carboxanilide).

**Table 3:** Selection of studies assessing morphological changes in human oral cells during cytotoxicity testing.

Tested Agent	Type of Cells	Results	Reference
Dental materials			
Cold-curing acrylic resin	Human periodontal ligament fibroblasts	The cells became rounder because of cytoplasmic shrinkage, and vacuolization was stimulated.	[108]
Dental adhesive	Human gingival fibroblasts	The fusiform-shaped cells eventually became detachable and round.	[33]
Materials for prosthetic components (polyether-ether-ketone and stainless steel)	Human gingival keratinocytes and fibroblasts	The morphology of both cell lines was not affected.	[109]
Titanium discs	Human gingival fibroblasts	The surface roughness of the titanium discs could influence cell morphology.	[110]
Discs with Laser-Lok (a laser-modified titanium surface), zirconia, and titanium surfaces	Human gingival fibroblasts	Cell morphology was different for the three materials; in the Laser-Lok group, the cells were elongated and had pseudopods, while in the zirconia group, they were round.	[111]
Silver tungstate (α-Ag_2_WO_4_) microcrystals	Human gingival fibroblasts	A normal morphology was observed after exposure to α-Ag_2_WO_4_ (7.81 μg/mL); a concentration of 78.1 μg/mL caused membrane disruption and complete cell death.	[62]
Citric acid (root surface demineralization, smear layer removal, dentin etching)	Human dental pulp cells	Cell retraction and cell surface blebbing were observed.	[112]
Endodontic sealers	Human gingival fibroblasts	Compromised cell membranes and loss of cell content were observed.	[113]
Hydrogen peroxide bleaching gels	Human dental pulp cells	Alterations in cell morphology appeared (e.g., shrinkage of the cytoplasm), proportional to the peroxide concentration.	[37]
Topical fluoride varnishes	Human gingival fibroblasts	Changes in cell morphology and in the actin cytoskeleton structure were observed.	[83]
Methacrylate-based CAD/CAM milled and 3D-printed samples	Human gingival fibroblasts	Apoptotic features (cell shrinking, membrane blebbing, and apoptotic body formation) were identified in fibroblasts incubated with methacrylate-based 3D samples.	[114]
Drugs/oral healthcare formulations			
ZnO, TiO_2_, SiO_2_, hydroxyapatite—bulk particle-sized materials and nanomaterials	Oral epithelial keratinocytes	Cells exposed to ZnO (bulk and nanomaterials) presented with reduced/absent filament network on cell surfaces, reduced diameter, and blebbing.	[32]
Chlorhexidine	Human gingival fibroblasts	Low concentrations of chlorhexidine did not influence cell morphology; high concentrations caused the appearance of small, round-shaped cells.	[71]
Valproic acid	Human gingival fibroblasts	High concentrations of valproic acid (8 mM) affected cell shape and proliferation.	[115]
Tacrolimus	Human gingival fibroblasts	Only at 100 µg/mL, the cell morphology was affected.	[116]
Zoledronic acid	Human gingival fibroblasts	Altered cell morphology and the presence of round-shaped cells with disrupted cytoplasmic membrane and numerous flake structures were observed.	[28]
Other agents			
Cigarette smoke and e-vapor condensates	Human gingival fibroblasts	The e-vapor condensates (nicotine-rich and nicotine-free) and cigarette smoke caused alterations in cell shape.	[40]
Traditional cigarette smoke and e-cigarette aerosol	Human epithelial gingival keratinocytes	Apoptotic features were detected after exposure (apoptotic cellular bodies, nuclear condensation, and DNA fragmentation).	[39]
E-cigarette vapors	Human gingival epithelial cells	The morphology of cells was altered; the small cuboidal cells changed into large, elongated shapes.	[58]
Metallic ions (beryllium (Be^+2^), chromium (Cr^+6^ and Cr^+3^), nickel (Ni^+2^), and molybdenum (Mo^+6^))	Human gingival fibroblasts	Hexavalent chromium and nickel induced irregularly shaped nuclei; all metal ions reduced the number of polyribosomes and the size of mitochondria; and beryllium and molybdenum caused pseudopodia.	[117]
Zinc oxide nanoparticles	Human gingival fibroblasts	Only at concentrations above 50 μg/mL did ZnO nanoparticles have altered cell morphology.	[57]
Chitosan-coated and alginate-coated liposomes	Human oral keratinocytes	Exposed cells did not exhibit an altered morphology at visual inspection.	[27]
Asiasari radix extracts	Human stem cells derived from the gingiva	Alterations in the cytoskeletal organization were observed at high concentrations of extracts (100 and 1000 μg/mL).	[118]
Areca nut products	Human gingival fibroblasts	Reduced cytoplasmic volume and shrinkage of the actin cytoskeleton were observed.	[61]
Er:YAG laser irradiation	Human gingival fibroblasts	Transient alterations of the mitochondria and ribosomal endoplasmic reticulum were observed.	[48]

Abbreviations: Er:YAG (erbium-doped yttrium–aluminum–garnet).

**Table 5:** Selection of studies assessing oxidative stress in human oral cells during cytotoxicity testing.

Investigated Parameter	Type of Cells	Tested Agent	Results	Reference
Dental materials				
Intracellular ROS/RNS levels mRNA expression of redox-regulated proteins	Human oral keratinocytes	Photoinitiators BAPO and TPO	No increase in the intracellular ROS/RNS was observed; BAPO modified the expression of oxidatively regulated enzymes.	[30]
ROS generation Expression of antioxidant enzymes	Human pulp- derived cells	Photoinitiators (CQ and TPO)	Adaptive changes in the expression of antioxidant enzymes were present.	[145]
Intracellular ROS levels	Human oral keratinocyte	Silver–palladium–gold–indium dental alloy	Increasing indium concentration was associated with increased intracellular ROS production.	[47]
Intracellular ROS levels	Human gingival fibroblast	Silver tungstate microcrystals	Only the highest tested concentration (78.1 μg/mL) caused an overproduction of ROS.	[62]
Intracellular ROS levels	Human pulp cells	Dental composites	The components released from composites caused cytotoxicity via ROS formation.	[149]
Intracellular GSH levels Lipid peroxidation	Human gingival fibroblasts	TEGDMA	The depletion of intracellular GSH was drastic after TEGDMA exposure. Signs of lipid peroxidation were recorded.	[90]
Intracellular GSH levels	Human oral keratinocytes	TEGDMA	Moderate ROS formation was recorded.	[135]
Nicotine and ENDS products				
Intracellular ROS levels	Cementoblasts	Nicotine	The levels of intracellular ROS increased after nicotine exposure in a time-dependent manner.	[150]
Intracellular GSH levels Expression of redox-regulated proteins	Normal human oral keratinocytes	Electronic cigarette aerosol	A significant decrease in intracellular GSH levels was recorded. The aerosols induced the expression of heme oxygenase 1.	[64]
Intracellular ROS levels	Human gingival fibroblasts	E-cigarette fluids	The production of ROS was increased by both nicotine-containing and nicotine-free fluids.	[151]
ROS generation	Human oral fibroblasts	Cigarette smoke and e-vapor condensates	After traditional cigarette smoke exposure, the formation of ROS was significantly higher than for e-vapor condensates.	[81]
ROS generation Lipid peroxidation	Oral epithelial cells	E-cigarette aerosols	Most of the tested e-liquids induced significant levels of oxidative stress, while lipid peroxidation was less common.	[54]
Immunodetection of protein carbonylation	Human periodontal ligament fibroblasts	E-cigarette vapors with flavorings	E-cig aerosols and flavorings caused carbonyl stress.	[138]
Other agents				
Intracellular GSH levels Generation of H_2_O_2_ in the cell culture medium	Human gingival fibroblasts	Theaflavin mixture purified from black tea	A pro-oxidant activity of the theaflavin mixture was observed, with a reduction in GSH levels (but this action was more intense for malignant cells).	[53]
Intracellular ROS levels	Human gingival fibroblast	ZnO nanoparticles	High concentrations significantly increased ROS levels, while low concentrations had no influence.	[57]
Intracellular ROS levels	Human gingival fibroblasts	Curcumin	Curcumin determined the dose-dependent production of ROS up to 15 μM, where it reached a plateau.	[85]
Intracellular ROS levels	Normal human oral fibroblasts	Areca nut	Cytokine secretion increased and triggered the generation of ROS.	[152]

Abbreviations: CQ (camphorquinone); ENDS (electronic nicotine delivery systems); GSH (glutathione); TEGDMA (triethylene glycol dimethacrylate); and TPO (diphenyl(acyl) phosphine oxide).

**Table 6:** Selection of studies assessing the inflammatory response in human oral cells during cytotoxicity testing.

Investigated Parameter	Type of Cells	Tested Agent	Results	Reference
Dental materials				
Gene expressions of IL1β, IL6, IL8, IL10, TNFα, and VEGF	Human gingival stroma fibroblasts	Dental adhesives	The adhesives upregulated the gene expression of IL1β, IL6, IL8, and VEGF, but did not affect the IL-10 and TNFα expression.	[33]
1L-6 and 1L-8 levels	Human gingival fibroblasts	Denture base acrylic resins	An increased secretion of cytokines was observed.	[158]
IL-1b, IL-6, IL-10, and TNF-α levels	Human dental pulp cells	Dental adhesives (the influence of HEMA and solvent concentrations)	HEMA 20% + ethanol significantly increased cytokine release (IL-6, IL-10, and TNF-α) after a 24 h exposure.	[159]
IL-6 and PGE2 levels	Human gingival fibroblasts and human oral mucosal keratinocytes	Three-dimensional-printed oral appliances	The PGE2 concentration in the gingival fibroblast cultures was reduced after exposure to some printable resins.	[155]
Nicotine, ENDS and tobacco products				
PGE2 and COX-2 levels IL 8 level	Human periodontal ligament fibroblasts	E-cigarette vapors with flavoring	E-cig aerosols and flavorings increased pro-inflammatory cytokine release and COX-2 levels.	[138]
Gene expressions of IL-1α, IL-1β, and IL-6	Human epithelial oral cells	E-cigarette liquid aerosols and traditional cigarette smoke	E-cig aerosol and traditional cigarette smoke increased the expression of interleukins (higher for traditional cigarette smoke).	[39]
Gene expressions of IL6, IL8, and TNFα	Human gingival fibroblasts	Flavored nicotine pouches and CRP1.1 (a smokeless tobacco reference product—snus)	Only the expression of the gene IL6 was upregulated by CRP1.1.	[160]
Other agents				
Production of IL-1β, IL-6, IL-8, IL-15, and TNF-α	Human gingival fibroblasts	Ethanolic extract of propolis and CAPE	CAPE inhibited the production of TNF-α and IL-6, caused by the LPS and IFN-α stimulation. The propolis extract did not significantly influence the concentrations of IL-1β and TNF-α.	[161]
PGE2 levels Gene expression of COX-2, IL-6, MMP-2, MMP-8, MIP-1α, and SDF-1	Human gingival fibroblasts	*Ecklonia cava* extract	The extract decreased the expression of pro-inflammatory enzymes and chemokines and reduced PGE2 production.	[162]
PGE2, NO, IL-6, and IL-8 levels	Human gingival fibroblasts	LPS from *Porphyromonas gingivalis* and asiatic acid	Asiatic acid reduced LPS-induced production of PGE2, IL-6, and IL-8 in a concentration-dependent manner.	[163]
Production of IL-1β and IL-6	Human gingival fibroblasts	Bamboo salt	Bamboo salt exhibited anti-inflammatory activities and inhibited the release of IL-1β and IL-6.	[164]
Production of IL-6 and IL-8	Human gingival fibroblasts	Ozone ointment	The ozone ointment inhibited the production of IL-6 and IL-8, induced by LPS.	[165]
Concentrations of TNF-α, IL-6, IL-8, MCP-1, and PGE2	Human gingival fibroblasts	Synthetic azulene- related compounds	Benzo[b]cyclohepta[e][1,4] thiazine was the most potent inhibitor of IL-8 production.	[166]

Abbreviations: CAPE (caffeic acid phenethyl ester); COX-2 (cyclooxygenase-2); HEMA (2-hydroxylmethyl methacrylate); LPS (lipopolysaccharides); IL (interleukin); MMP (matrix metalloproteinases); MIP (macrophage inflammatory proteins); SDF-1 (stromal cell-derived factor 1); TNFα (tumor necrosis factor α); and VEGF (vascular endothelial growth factor).

**Table 8:** Selection of studies using organ-on-a-chip models for toxicity assessment.

Type of Organ-on-a-Chip	Investigated Parameter	Tested Agent	Results	Reference
Tooth-on-a-chip	Cytotoxicity, morphology, proliferation, and metabolic activity	Dental materials (HEMA, phosphoric acid, Adper-Scotchbond)	All dental materials had cytotoxic effects and affected morphology and metabolic activity.	[207]
	Cell viability and morphology	Silver diamine fluoride	Silver diamine fluoride toxicity against dental pulp stem cells depended on the thickness of the dentin.	[203]
Pulp-dentin-on-a-chip	Cell viability, morphology, pH, and release of TGFβ	Calcium silicate cements	Of the three tested compounds, one compound affected cell proliferation and morphology, while the other two promoted proliferation and the release of TGFβ.	[211]
Oral-mucosa-on-a-chip	Cell viability, morphology, and organization	HEMA	HEMA reduced cell viability and disrupted the mucosal layer organization.	[212]
	Cytotoxicity and morphology	HEMA	HEMA reduced mucosal cell viability.	[213]
Gingiva-on-a-chip	Cell viability and cytotoxicity	Oral-care formulations (mouthwashes)	The mucosal irritation potential was slightly higher for alcohol-based mouthwashes.	[204]

Abbreviations: HEMA (2-hydroxylmethyl methacrylate); and TGFβ (transforming growth factor–β).

## Abbreviations

The following abbreviations are used in this manuscript:

5-CFDA-AM	5-carboxyfluorescein diacetate acetoxymethyl ester
3H-TdR	tritiated thymidine
8OHdG	8-hydroxy-2′-deoxyguanosine
8-oxo-Gua	8-oxoguanine
ATP	adenosine triphosphate
BAPO	phenyl-bis(acyl) phosphine oxide
BAPP	2,2-bis[4-(acryloxypropoxy) phenyl] propane
BAX	Bcl-2-associated X protein
BisGMA	bisphenol A-glycidylmethacrylate
BrdU	5-bromo-2′-deoxyuridine
C	catechin
CAPE	caffeic acid phenethyl ester
CAT	catalase
CG	catechin gallate
CLFPs	cytoplasm-localized fluorescent probes
CPD	cyclobutane pyrimidine dimer
CQ	camphorquinone
CRP1.1	a smokeless tobacco reference product—snus
CSFs	colony-stimulating factors
DCF	2′,7′-dichlorofluorescein
DPIC	diphenyliodonium chloride
DNPH	2,4-dinitrophenylhydrazine
EC	epicatechin
ECG	epicatechin gallate
EGC	epigallocatechin
EGCG	epigallocatechin gallate
ENDS	electronic nicotine delivery systems
EdU	5-ethynyl-2-deoxyuridine
Er:YAG	erbium-doped yttrium aluminum garnet
FACS	fluorescence-activated cell sorting
G6PD	glucose-6-phosphate dehydrogenase
GPx	glutathione peroxidase family
GR	glutathione reductase
GSH	glutathione
H2DCF	2′,7′-dichlorodihydrofluorescein diacetate
HEMA	2-hydroxy-ethyl-methacrylate
IWGT WG	the International Workshop on Genotoxicity Testing Working Group
LDH	lactate dehydrogenase
LPS	lipopolysaccharides
MDA	malondialdehyde
MIP	macrophage inflammatory proteins
MMPs	matrix metalloproteinases
MTS	dimethylthiazolcarboxymethoxyphenylsulfophenyl-tetrazolium
MTT	methylthiazolyldiphenyltetrazolium bromide
NADPH	nicotinamide adenine dinucleotide phosphate
NRU	neutral red uptake
PARP1	poly-(ADP-ribose) polymerase-1
PCNA	proliferating cell nuclear antigen
PHH3	phosphohistone H3
RAGE	receptor for advanced glycation end
RIPK1	receptor interacting serine/threonine kinase 1
SAP130	Sin3A associated protein 130 kDa
SDF-1	stromal cell-derived factor 1
SOD	superoxide dismutase
SRB	sulforhodamine B
TBA	thiobarbituric acid
TEGD-MA	triethyleneglycol-dimethacrylate
TEEGDMA	tetraethyleneglycol dimethacrylate
TEER	transepithelial electrical resistance
TGFs	transforming growth factors
TNFα	tumor necrosis factor α
TPO	diphenyl(acyl) phosphine oxide
TPSB	triphenyl-stibane
TPP	triphenylphosphane
UDMA	urethane dimethacrylate
VEGF	vascular endothelial growth factor
WST	water-soluble tetrazolium salt
XTT	methoxynitrosulfophenyl-tetrazolium carboxanilide
